# A Functional Regulatory Variant of *FGF9* Gene Affected the Body Weight in Hu Sheep

**DOI:** 10.3390/ani15162375

**Published:** 2025-08-13

**Authors:** Xiaoxue Zhang, Deyin Zhang, Fadi Li, Dan Xu, Jiangbo Cheng, Xiaolong Li, Yuan Zhao, Yukun Zhang, Liming Zhao, Peiliang Cao, Huibin Tian, Weiwei Wu, Weimin Wang

**Affiliations:** 1 College of Animal Science and Technology, Gansu Agricultural University, Lanzhou 730070, China; zhangxx@gsau.edu.cn (X.Z.); c3264887984@outlook.com (P.C.); 2 State Key Laboratory of Herbage Improvement and Grassland Agro-Ecosystems, Key Laboratory of Grassland Livestock Industry Innovation, Ministry of Agriculture and Rural Affairs, Engineering Research Center of Grassland Industry, Ministry of Education, College of Pastoral Agriculture Science and Technology, Lanzhou University, Lanzhou 730020, China; zdy1213@163.com (D.Z.); lifd@lzu.edu.cn (F.L.); 15045093462@163.com (D.X.); 15117098920@163.com (J.C.); lixllil@163.com (X.L.); zhaoyuan_10@163.com (Y.Z.); 120220900701@lzu.edu.cn (Y.Z.); zlmfxy1807285865@163.com (L.Z.); tianhb@lzu.edu.cn (H.T.); 3 Institute of Animal Science, Xinjiang Academy of Animal Sciences, Urumqi 830011, China; wuweiweigp@foxmail.com

**Keywords:** *FGF9* gene, body weight, SNP, sheep, epigenetic, hypophysis

## Abstract

Body weight (BW) is a critical economic trait closely linked to livestock meat production performance and producer profitability. In the present study, we measured individual BW of 1070 male Hu sheep at six growth stages (80, 100, 120, 140, 160, and 180 days of age) and conducted descriptive statistical analyses. Results showed that the coefficient of variation (CV) for BW at each stage was higher than 13%. Additionally, we investigated the expression patterns of the *FGF9* gene and its associations with single nucleotide polymorphisms (SNPs) and BW. Quantitative real-time PCR (qRT-PCR) revealed *FGF9* is significantly higher expressed in the hypophysis tissues that in the other tested tissues. Association analyses indicated that the SNP *FGF9*:c.382-1264C>T was significantly associated with BW across different measurement periods. Finally, we performed assay for transposase-accessible chromatin using sequencing (ATAC-seq) and cleavage under targets and tagmentation (CUT&Tag) techniques to identify the OCRs (open chromatin regions) and regulatory regions in the hypophysis tissues of Hu sheep, and we obtained an average of 221243, 52692, and 20957.5 peaks in the ATAC-seq, H3K27ac, and H3K4me3 data per sample. Simultaneously, by integrating the SNPs of *FGF9* and hypophysis tissue’s epigenomic data, we found that the SNP *FGF9*:c.382-1264C>T was located in the adjacent OCR- and H3K27ac-modified peaks. Therefore, we propose that the SNP *FGF9*:c.382-1264C>T plays an important role in regulating body weight in sheep. Overall, this study generated a valuable epigenetic dataset (ATAC-seq, H3K27ac, and H3K4me3) in sheep hypophysis tissue and identified a novel functional regulatory variant for improving body weight in sheep.

## Simple Summary

Body weight is a critical economic indicator. In this study, we collected the body weight data of a large homogeneous population of sheep, and the single nucleotide polymorphisms of *FGF9* gene were verified and analyzed. Additionally, we identified regulatory elements in the hypophysis tissue by CUT&Tag and ATAC-seq methods and found that the functional regulatory variant can control body weight by integrating the results of single nucleotide polymorphisms and epigenomic data. This study generated a valuable epigenetic dataset (ATAC-seq, H3K27ac, and H3K4me3) in sheep hypophysis tissue and provided a novel functional variant for understanding of body weight variation in sheep.

## 1. Introduction

Sheep, as small ruminants, are widely raised globally, providing a critical source of dairy products, meat, and wool for human consumption and playing an indispensable role in the agricultural economy [1,2]. In the sheep industry, body weight is a key economic trait, closely linked to mutton production efficiency, the economic benefits of husbandry enterprises, and breeders’ enthusiasm [2,3]. Increasing body weight is an effective strategy to enhance meat performance and profitability for producers. Improving animal meat performance is a primary goal in most breeding programs under China’s National Genetic Improvement Plan for Livestock and Poultry (2021–2035). Body weight is a complex trait regulated by several major genes and numerous minor genes [4]. Additionally, with the rapid advancement of molecular genetics and sequencing technologies, molecular breeding has been applied to the genetic improvement of livestock and poultry. Compared with traditional breeding methods, it not only accelerates genetic progress but also reduces costs. Simultaneously, the implementation of the Functional Annotation of Animal Genomes (FAANG) consortium and Farm Animal GTEx (FarmGTEx) has provided valuable data for identifying causative variants underlying important traits in livestock [5,6]. However, to date, only a limited number of major genes and molecular markers associated with body weight have been identified, and the underlying genetic mechanisms remain incompletely understood. Therefore, identifying key genes and molecular genetic markers for body weight traits in sheep is crucial, with the aim of advancing marker-assisted selection.

In the present study, we selected the *FGF9* gene as a candidate gene based on our previous genome-wide selection signature analysis of indigenous and improved sheep breeds using whole-genome resequencing data [7]. FGF9 is a member of the fibroblast growth factor (FGF) family that mainly binds receptors FGFR2 and FGFR3 [8,9] and plays a crucial role in regulating cell growth, embryonic development, skeletal development [10,11], energy metabolism, and obesity [12,13]. For example, Sun et al. found that *FGF9* acts as an inhibitory factor in the browning of white adipocytes and is associated with obesity in mice and humans [12]. In chickens, studies have indicated that *FGF9* is involved in lipid formation and regulation [14] and plays a vital role in progesterone production in hierarchical granulosa cells [15]. In pigs, Wang et al. reported that the *FGF9* gene may be involved in the regulation of embryo implantation [16]. In sheep, the existing literature has shown that the *FGF9* gene plays an important role in early gonadal development and hair follicle growth and development [17,18]. Additionally, Li et al. identified an association between the *FGF9* gene and weaning weight in sheep through genome-wide association study [19]. Hu sheep is a unique Chinese sheep breed known for its high rearing rate, along with traits such as high fecundity, early maturity, and adaptability to housed feeding. However, the breed exhibits lower growth rates, which limits its production efficiency in the livestock industry.

Hence, the aim of this study is to analyze the expression characteristics of the *FGF9* gene in various tissues of 6-month-old Hu sheep and to identify the functional regulatory variants of the *FGF9* gene that affect sheep body weight by integrating epigenomic data.

## 2. Materials and Methods

### 2.1. Collection of Phenotypic Data and Samples

In total, 1070 male Hu lambs with detailed birth information were randomly selected as the experimental population. These lambs were sourced from large-scale Hu sheep farms, namely Lanzhou Tianxin Sheep Industry Co. Ltd. (Lanzhou, China), Changxing Yongsheng Husbandry Co. Ltd. (Huzhou, China), Shandong Runlin Sheep Industry Co. Ltd. (Linqing, China), Hangzhou Pangda Agricultural Development Co. Ltd. (Hangzhou, China), Jinchang Zhongtian Sheep Industry Co. Ltd. (Jinchang, China), and Zhongsheng Huamei Sheep Industry Development Co. Ltd (Qiangyang, China). After weaning at 56 days old, they were transferred to Minqin Defu Agriculture Co. Ltd (Wuwei, China). and raised in the same feeding regime and management conditions until they were 180 days old. Throughout the experiment, all lambs had free access to water and food and were weighed and their weight recorded in the morning before feeding at 80, 100, 120, 140, 160, and 180 days using a calibrated electronic scale. Meanwhile, blood samples were collected from each sheep by puncturing the jugular at 180 days of age. We used tubes containing heparin sodium as an anticoagulant, and blood samples were stored at −20 °C until DNA extraction. After slaughter, 36 samples were collected for tissue expression profile analysis from nine tissues (hypophysis, liver, longissimus dorsi, lymph, rumen, spleen, kidney, heart, and tail fat) of four 6-month-old Hu sheep that were randomly selected and had similar body weights. In addition, we selected two half-sib male Hu lambs aged 6 months to collect the hypophysis tissue samples, which were cleaned with phosphate-buffered saline (PBS) and then transferred into 2 mL cryogenic vials for ATAC-seq and CUT&Tag.

### 2.2. DNA, RNA Extraction and Quality Control

Genomic DNA were isolated from 1070 blood samples using an EasyPure Blood Genomic DNA Kit (TransGen Biotech, Beijing, China) in accordance with the manufacturer’s instruction. Total RNA was extracted for 36 samples from nine tissues (hypophysis, liver, longissimus dorsi, lymph, rumen, spleen, kidney, heart, and tail fat) of four sheep with TRIzol reagent (TransGen, Beijing, China) according to the manufacturer’s instruction. After extraction, the DNA and RNA quality were checked by using NanoDrop 2000 spectrophotometer (Thermo Scientific, Waltham, MA, USA) and agarose gel electrophoresis. Finally, the DNA samples were diluted to 40 ng/μL and stored at −20 °C for PCR amplification and SNP genotyping. The total RNA was used for subsequent quantitative real-time PCR (qRT-PCR) analysis.

### 2.3. SNP Identification and Genotyping

To verify the polymorphisms of *FGF9* gene in Hu sheep population, we firstly converted the coordinates of the three sites (NC_019467.2:g.35570188A>G, NC_019467.2:g.35570233T>C, and NC_019467.2:g.35571510C>T) from Oar_v4.0 to Oar_rambouillet_v1.0 (*FGF9*:c.438A>G, *FGF9*:c.393T>C, and *FGF9*:c.382-1264C>T) by sequence alignment (analysis performed in December 2022). Secondly, primer pairs containing these three SNPs and other exon regions were designed with Oligo 7.0 software using the Oar_rambouillet_v1.0 genomic sequences. Detailed information of the primers is shown in Supplementary Table S1. The PCR reaction volume and conditions were as described in our previous study [20], and the PCR products were qualified by agarose gel electrophoresis and then sequenced using the Sanger sequencing method in Tsingke (Xi’an, China) with the primer the same as the amplification primer. Finally, the SNPs were genotyped using the Sequenom MassARRAY^®^ SNP technique (Beijing Compass Biotechnology Co., Ltd., Beijing, China) and competitive allele-specific FRET-based PCR (KASPar) assays (LGC Genomics). The PCR primers of genotyping were designed and are listed in Supplementary Table S2, and the detailed information about the system and procedures were described in a previously published method [21,22].

### 2.4. Quantitative Real-Time PCR Analysis

First-strand cDNA was generated from 200 ng of total RNA using an Evo M-MLV RT Kit with gDNA Clean for qPCR (Accurate Biotechnology Co., Ltd., Changsha, China) as recommended by the manufacturer and was stored at −20 °C. The relative expression of the *FGF9* gene was determined in nine tissues of four sheep by qRT-PCR. Specific primers were designed using Oligo 7.0 software and were synthesized by Tsingke (Xi’an, China). The qRT-PCR was performed on CFX384 system (Bio-Rad, Hercules, CA, USA) and SYBR Green Premix Pro Taq HS qPCR Kit (Accurate Biotechnology Co., Ltd., Changsha, China); *β-actin* was used as the endogenous reference gene. The qRT-PCR procedure adopted the two-step method, which was as follows: 95 °C for 30 s, 40 cycles of 95 °C for 5 s, and 60 °C for 30 s. The relative expression levels of *FGF9* gene in nine tissues were calculated using 2^−ΔΔCt^ method. Primer sequences are listed in Table 1.

### 2.5. ATAC-Seq and CUT&Tag Library Construction and Sequencing

In this study, ATAC-seq and CUT&Tag experiments were completed in Wuhan Yingzi Gene Technology Co., Ltd. (Wuhan, China). The sequencing library was compiled following a previously described method with slight modifications [23,24]. Briefly, the frozen tissue sample was crushed in liquid nitrogen and homogenized into cell suspensions with 1 mL ice-cold PBS. The nuclei were isolated and incubated with the Tn5 transposase reaction mixture at 37 °C for 1 h. The library was then purified and amplified using DNA Purification and Concentration Kit (Genstone Biotech, TD413, Sydney, Australia) and NEBNext High-Fidelity 2X PCR Master Mix (NEB, M0541L) in accordance with the manufacturer’s instruction, followed by purification using KAPA Pure Beads (Kapa Biosystems, KS8002). Finally, the Illumina HiSeq X Ten paired-end 150 bp (PE150) platform was used for sequencing. For CUT&Tag, the nuclei were isolated and combined with concanavalin A-coated magnetic beads (BioMag Plus, BP531, Fishers, IN, USA) for 20 min at room temperature. Subsequently, the cell magnetic beads were combined with the corresponding antibodies (H3 lysine 4 trimethylation: H3K4me3: 04-745, Millipore; H3 lysine 27 acetylation: H3K27ac: ab4729, Abcam; and IgG: AC005, ABclonal, Woburn, MA, USA). After 1 h incubation at room temperature, the primary antibody was removed and incubated with a secondary antibody (goat anti-rabbit IgG: ab6702, Abcam, Cambridge, UK) for 1 h at room temperature, followed by three washes with Dig-Wash Buffer. Next, the protein G-Tn5 complex was added and incubated for 1 h at room temperature and was washed using 1× Dig-300 Buffer (Vazyme, TD901-TD902, Nanjing, China) and incubated in Mg^2+^ activation system (Invitrogen™, AM9530G, Waltham, MA, USA) in 37 °C for 1 h. Finally, the sodium dodecyl sulfate (SDS) buffer (Invitrogen™, 15553-027) was used to stop tagmentation reaction, the Tagment DNA Extract Beads (Novoprotein, N245) were used to extract DNA, and amplification was performed using NEBNext High-Fidelity 2X PCR Master Mix and adapting sequencing connector primers. Finally, the PCR product was purified with the KAPA Pure Beads and sequenced on an Illumina HiSeq X Ten PE150.

### 2.6. ATAC-Seq and CUT&Tag Data Analysis

For ATAC-seq and CUT&Tag analysis, two biological replicate samples were included for each assay, and each replicate was processed and analyzed independently. The raw reads were filtered using fastp (version 0.23.1) software with default parameters. After trimming, the quality of the reads was assessed using FastQC (version 0.11.9) software. The clean reads were mapped onto the sheep reference genome (Oar_rambouillet_v1.0) with Bowtie2 (version 2.2.4) software, and the mapping data were then filtered with Samtools (version 1.12) and Picard (version 3.1.1) software. Finally, the peak was called using MACS2 (version 2.2.6) with the parameter -g 2869914396 -p 0.01 --nomodel --shift -75 --extsize 150 -B --SPMR --keep-dup all --call-summits. The peak was annotated by using the ChIPseeker package [25]. The BEDTools intersect -wa -wb was used to obtain the intersection of SNP and peak. The results were visualized using IGV (version 2.14.1) browser.

### 2.7. Statistical Analysis

The descriptive statistics analysis for phenotypic data of body weight traits was conducted using the describe () function from the psych package in R software. For analyzing single nucleotide polymorphism (SNP)-related parameters, including allele frequencies, genotypic frequencies, and population genetic indices [polymorphic information content (PIC), expected heterozygosity (He), and Hardy–Weinberg equilibrium (HWE) tests], we utilized two approaches: an online tool (http://www.msrcall.com/Gdicall.aspx, accessed on 15 June 2022) and the SNPassoc package in R (version 4.1.1).

To explore the association between *FGF9* genotypes and body weight traits, a general linear model (GLM) was employed. The model was structured as follows:

Y_ij_ = μ + Genotype_i_ + Farm_j_ + ε_ij_

In this model, Y_ij_ denotes the phenotypic observation value of body weight traits, μ represents the overall population mean; Genotype_i_ is the fixed effect of the ith genotype; Farm_j_ is the effect of the jth farm; ε_ij_ indicates random error. Statistical significance was determined at a threshold of *p* < 0.05. For pairwise comparisons of body weight among different genotypes, the Tukey HSD test was conducted, and all phenotype values are presented as mean ± standard deviation (SD).

## 3. Results

### 3.1. Descriptive Statistics for Body Weight Traits

In the present study, the individual body weight (BW) of male Hu lambs (*n* = 1070) was measured and recorded at different growth stages. The abbreviated name of body weight traits as well as the descriptive statistics of phenotype data are provided in Table 2. The results showed that the mean values for body weight of six periods were 19.42 kg, 24.55 kg, 30.37 kg, 36.07 kg, 41.87 kg, and 46.80 kg, respectively. The coefficient of variation (CV) of body weight at each stage was higher than 13%, and the CV for the weight of BW80 was the highest to 20.73%. In addition, the phenotypic values for body weight of six periods are approximately normally distributed with small kurtosis (−0.061 to 0.242) and skewness (−0.009 to 0.245) values, as their absolute values were less than 1.

### 3.2. Expression Features Analysis of FGF9 Gene in Sheep

**Figure 1:**
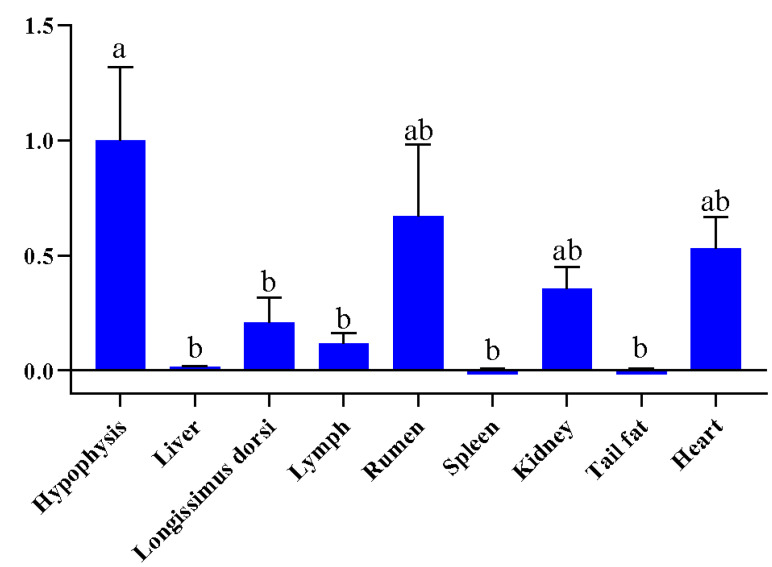
Relative expression of *FGF9* mRNA in different tissues of the Hu sheep. Different lowercase letters indicate significant differences (*p* < 0.05).

The quantitative RT-PCR was performed to investigate the expression features of *FGF9* gene in different tissues (hypophysis, liver, longissimus dorsi, lymph, rumen, spleen, kidney, heart, and tail fat), and the results indicated that the *FGF9* gene was widely expressed in tested tissues, with the highest expression level detected in the hypophysis; in contrast, the spleen, tail fat, and liver showed relatively lower expression levels compared to other tissues, as shown in Figure 1.

### 3.3. Detection and Genotyping of FGF9 Polymorphism in Hu Sheep

**Figure 2:**
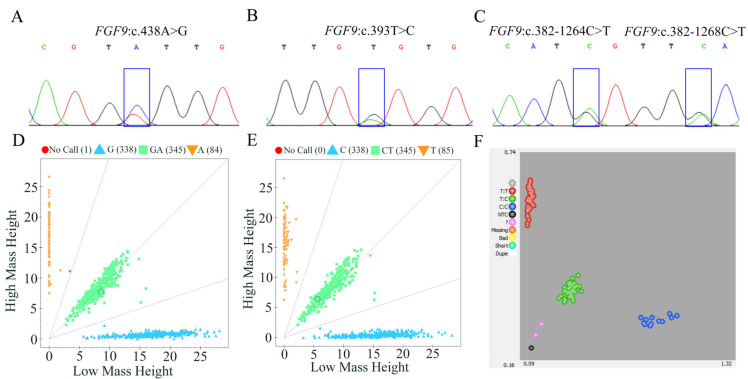
Verification and genotyping of SNPs of *FGF9* gene in sheep. (**A**,**D**) Sequence chromatogram (**A**) and Sequenom MassARRAY^®^ genotyping (**D**) for the SNP *FGF9*:c.438A>G. (**B**,**E**) Sequence chromatogram (**B**) and Sequenom MassARRAY^®^ genotyping (**E**) for the SNP *FGF9*:c.393T>C. (**C**,**F**) Sequence chromatogram (**C**) and KASP genotyping (**F**) for the SNP *FGF9*:c.382-1264C>T.

In our previous study, genome-wide selection tests comparing landraces and improved sheep breeds revealed strong selection signals in the genomic region of *FGF9* [7]. In the present study, leveraging previously generated genomic data [7], we analyzed allele frequencies of these three variants across distinct populations. Results revealed that at the *FGF9*:c.438A>G locus, the A allele was detected at 0.73 in Chinese indigenous sheep, with the G allele at 0.27. Conversely, imported sheep exhibited a reciprocal pattern: the G allele predominated at 0.73, while the A allele was restricted to 0.27. For *FGF9*:c.393T>C, alleles T and C were skewed toward the T allele in Chinese indigenous sheep (0.73 and 0.27, respectively). In imported sheep, the C allele was significantly elevated to 0.72, with the T allele reduced to 0.28. For *FGF9*:c.382-1264C>T, the T allele occurred at 0.73 in Chinese indigenous sheep, with the C allele at 0.27. In imported sheep, the C allele predominated (0.71), while the T allele was restricted to 0.29 (Figure S1). To further investigate the polymorphism of the aforementioned SNPs and other exon regions of the *FGF9* gene in the Hu sheep population with accurately recorded body weight data, we amplified sequences containing these SNPs and other exon regions of the *FGF9* gene using specific PCR primer pairs (Supplementary Table S1), with DNA pools from 20 Hu sheep as templates. Sanger sequencing of the PCR products identified four SNPs in the *FGF9* gene (Figure 2). Among these, two SNPs were only 3 bp apart, so a total of three SNPs were selected for genotyping in the experimental population (Figure 2).

### 3.4. Population Genetic Parameter Analysis of SNPs in FGF9 Gene

Population genetic parameters analyses of three SNPs in *FGF9* gene were performed according to the genotyping data in the Hu sheep experimental population, and the results are shown in Figure S2. The results revealed that these three SNPs exhibited moderate polymorphism (polymorphism information content, PIC: 0.25 < PIC < 0.5) and were in Hardy–Weinberg equilibrium (HWE, *p* > 0.05). Furthermore, linkage disequilibrium analysis for the SNPs *FGF9*:c.438A>G and *FGF9*:c.393T>C, using the aforementioned genotyping data, showed that the two SNPs were in a state of strong linkage (Figure S3).

### 3.5. Association of Polymorphisms in FGF9 with Body Weight in Hu Sheep

The results of association analysis indicated that the SNP *FGF9*:c.438A>G was not significantly associated with body weight, while the SNP *FGF9*:c.382-1264C>T was significantly associated with body weight at six stages (Table 3). Moreover, except BW180, the body weight of Hu sheep at different periods with the *TT* genotype was the highest and was significantly higher than those in the animals with the *CC* genotype (*p* < 0.05).

### 3.6. Epigenetic Data (ATAC-seq, H3K4me3, and H3K427ac) Quality Control of Hypophysis Tissue in Sheep

To further explore the potential regulatory roles of the SNPs related to sheep body weight, we characterized open chromatin regions using assay for transposase-accessible chromatin using sequencing and also identified the genomic localization of histone H3K27ac and H3K4me3 by CUT&Tag methods in sheep hypophysis tissue. In total, 702,867,172; 160,076,532; and 201,741,220 raw reads were obtained in ATAC-Seq, H3K27ac, and H3K4me3, respectively. After filtration, 693,308,570; 149,604,394; and 181,906,606 clean reads were obtained. Comparison of these clean reads to the sheep reference genome (Oar_rambouillet_v1.0) revealed mapping rates ranging from 97.40% to 99.64%, and the average relative strand cross-correlation coefficient (RSC) and fraction of reads in peaks (FRiP) were 1.43 ± 0.25 and 0.55 ± 0.26, respectively (Table 4). These results showed our data could be used for subsequent analyses.

### 3.7. Comprehensive Profiling of Genetic Variants and Epigenomic Characteristics

**Figure 3:**
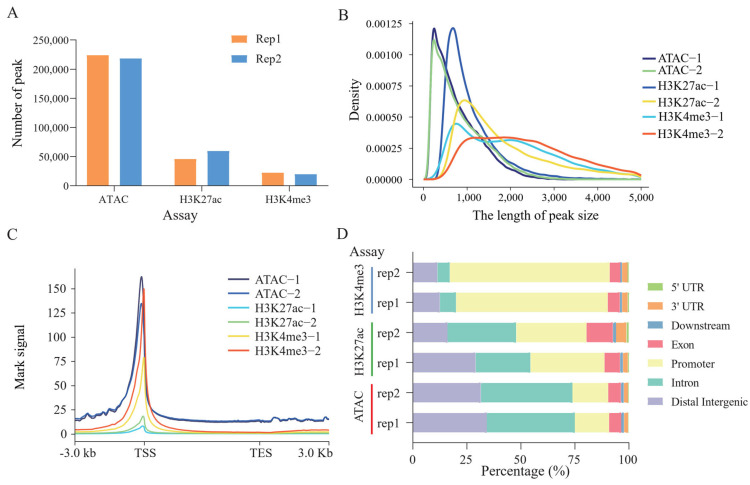
Characteristics of epigenetic data for hypophysis tissue in sheep. (**A**) Peak number of ATAC-seq, H3K27ac, and H3K4me3. (**B**) Peak length distribution of ATAC-seq, H3K27ac, and H3K4me3. (**C**) The distribution of epigenetic signals around TSSs of genes. TSS, transcription start site; TES, transcription end site. (**D**) Annotation and distribution of epigenetic mark peaks.

**Figure 4:**
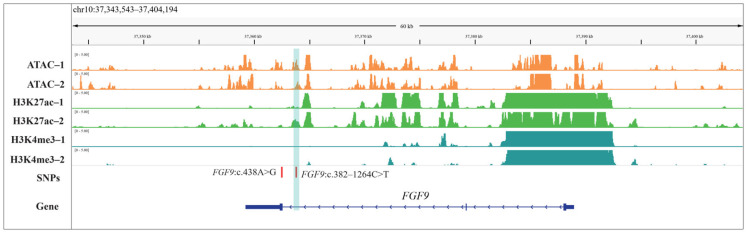
Epigenetic (ATAC, H3K27ac, and H3K4me3) signal on chromosome 10 containing *FGF9* gene. Orange tracks represent ATAC-seq signals, green tracks represent H3K27ac signals, and cyan tracks represent H3K4me3 signal; the same color represents a biological repetition of the same assay.

We obtained an average of 221,243; 52692; and 20,957.5 peaks in the ATAC-seq, H3K27ac, and H3K4me3 data (Figure 3A), with average lengths of 801.46, 1783.64, and 2467.03 bp, respectively (Figure 3B). Most OCRs were annotated to non-coding regions, mainly including introns (41.58%) and distal intergenic regions (32.98%), followed by promoter (16.19%), exons (5.77%), while H3K27ac and H3K4me3 peaks were mainly annotated to the promoter region (H3K27ac: 33.37%; H3K4me3: 72.15%) of the genes (Figure 3D). In addition, three epigenetic marks (ATAC-seq, H3K4me3, and H3K27ac) signals were more enriched near the transcription start sites (TSS) of genes (Figure 3C). Finally, we integrated the epigenetic data with the SNPs of *FGF9* gene, the results indicated higher OCR ~900 bp downstream of the SNP *FGF9*:c.382-1264C>T, and the SNP was also localized to the downstream peak (~700 bp) of H3K27ac modification, suggesting this polymorphism may influence *FGF9* gene expression by altering chromatin accessibility or transcription factor binding, whereas other SNPs were not located in the adjacent OCR- and H3K27ac-modified peaks (Figure 4). Therefore, we suspect that the SNP *FGF9*:c.382-1264C>T plays an important role in regulating body weight in sheep.

## 4. Discussion

Improving meat production is a primary objective in mutton sheep breeding. While previous studies have mapped 329 quantitative trait loci (QTLs) associated with body weight traits based on records in the Sheep QTL Database (released 27 December 2023; https://www.animalgenome.org/cgi-bin/QTLdb/OA/index), most candidate variants reside in non-coding genomic regions [26,27,28]; only a limited number of causative SNPs have been identified for body weight traits in sheep. Therefore, identifying functional regulatory variations plays an important role in enhancing body weight in current sheep breeding programs.

In the present study, we measured and recorded the body weight of male Hu lambs (n = 1070) from 80 to 180 days of age under the same feeding regime and management conditions. We found that body weight traits exhibit great potential for selection. These phenotypic data provide a valuable resource for identifying key genetic variations underlying body weight. Additionally, based on our previous studies, we selected the *FGF9* gene as a candidate gene for follow-up experiments. qRT-PCR results indicated that the relative expression of FGF9 was higher in the hypophysis than in other tested tissues; the hypophysis plays a central role in regulating growth and metabolism [29,30]. Previous studies have shown that *FGF9* acts as a regulator of bone development and identified that a *FGF9* loss-of-function mutation impairs early joint formation in a mouse model [31]. Additionally, Huang et al. found that *FGF9* inhibits the myogenic differentiation of C2C12 and human muscle cells, which suggests that *FGF9* may play an important role in modulating myogenesis [32]. In the present study, therefore, three SNPs of *FGF9* gene were genotyped in a Hu population with accurate body weight data records. Genotyping results revealed that these SNPs exhibited moderate polymorphism (0.25 < PIC < 0.5) and conformed to Hardy–Weinberg equilibrium (*p* > 0.05) in the experimental population. This indicates that the studied Hu sheep population harbors sufficient genetic variation and maintains a stable genetic structure, thereby laying a robust foundation for subsequent analyses of associations between these SNPs and body weight traits. Furthermore, based on data from our previous studies data, we found that the allele frequencies of these loci differ significantly between Chinese indigenous sheep breeds and imported sheep breeds [7]. This divergence is likely associated with the differences in growth rate, body weight, and other traits between indigenous and imported sheep breeds.

To further explore whether these variant loci are associated with body weight traits, we performed association analyses in the Hu sheep population. The results revealed that the SNP *FGF9*:c.382-1264C>T was significantly associated with body weight, with all BW phenotype values of animals carrying the *TT* and *TC* genotypes being significantly higher than those of animals carrying the *CC* genotype. In addition, in the experimental population, the *CC* genotype was rare (~11.8%; 118 individuals), while *TT* (~48.1%; 481 individuals) and *TC* (~46.8%; 468 individuals) dominated. Notably, *TT* and TC showed minimal phenotypic divergence, implying that T-allele carriers (*TT*/*TC*) uniformly confer growth advantages. Selecting for T alleles could thus improve weight gain, which is consistent with the high frequency of T-allele-bearing genotypes in the population. Additionally, Li et al. identified the *FGF9* gene as a key candidate gene influencing weaning weight in Hu sheep via a genome-wide association study [19], which supports our findings. Although this SNP was located in the intronic region, increasing evidence has indicated that non-coding SNPs can regulate enhancer activity, promoter activity, and the expression of distant target genes through spatial interactions—especially when they are localized in epigenetically active regions such as OCRs- or H3K27ac-modified peaks (a marker of active enhancers/promoters). For example, Pan et al. identified an SNP in these muscle-specific active strong enhancers, which may regulate the expression of *ALPK2* and serve as a candidate causal variant contributing to average daily gain in pig [33]. Zhu et al. demonstrated that the locus most significantly associated with 8-week-old body weight in chickens (position 170,526,091 bp within *CAB39L*) is localized to the OCR of the duodenum. This locus may exert regulatory effects on 8-week-old body weight (BW8) by modulating the transcriptional activity of *CAB39L* [34]. Smemo et al. found that the top SNP in the intronic region of the *FTO* gene association with obesity by regulating the expression of the *IRX3* and *IRX5* genes nearby [35]. Miao et al. identified two SNPs near the *BMP2* affecting the loin muscle depth in pig by integrating GWAS and 3D epigenomics [36]. Therefore, in this study, we identified the regulatory elements with ATAC-seq and CUT&Tag and performed integrated analysis with SNPs data; the result indicated the SNP *FGF9*:c.382-1264C>T was localized in the adjacent OCR- and H3K27ac-modified peaks. While OCRs are critical for enabling transcription factor binding and facilitating interactions between regulatory elements and gene promoters, H3K27ac is a well-characterized epigenetic marker of active enhancers—regions that drive transcriptional activation by looping to target gene promoters [37,38]. Together, these epigenetic signatures strongly suggest that this SNP resides within a functionally active genomic segment, where allelic variation could alter the accessibility of the chromatin landscape or disrupt the binding affinity of transcription factors or co-activators associated with H3K27ac-mediated enhancement. In addition, protein–protein interaction network analysis of *FGF9* revealed its interactions with fibroblast growth factor receptors (FGFR1/2/3/4), transforming growth factor beta-1 (TGFB1), SRY-box transcription factor 9 (SOX9), and bone morphogenetic proteins (BMP2/4), implying that *FGF9* may play multifaceted roles in developmental biology and the maintenance of tissue homeostasis. Therefore, these results suggest the functional regulatory variant may regulate *FGF9* expression and is an important candidate mutation affecting body weight in sheep. Nonetheless, further in-depth investigations into the relationships between genotype, gene expression, and body weight are warranted to validate and expand these findings.

## 5. Conclusions

Our study demonstrates the functional regulatory variant *FGF9*:c.382-1264C>T as a promising candidate marker for improving weight traits in sheep. However, to fully translate these findings into practical breeding applications, further investigations are required: firstly to clarify the functional mechanisms by which *FGF9* regulates weight traits and secondly to validate its utility across diverse sheep breeds. Such efforts will strengthen the practical applicability of these findings in breeding programs.

## Figures and Tables

**Table 1:** Details of primer sequences used for qRT-PCR.

Primer Name	Primer Sequence (5′-3′)	Annealing Temperature (°C)	Amplicon Size (bp)
*FGF9*-expression-F	GAAGCTGCATTTAATCCCAAG	60	204 bp
*FGF9*-expression-R	GATCACTTTTGGCTGTCTCC		
*β-actin*-F	TCCGTGACATCAAGGAGAAGC	60	256 bp
*β-actin*-R	CCGTGTTGGCGTAGAGGT		

**Table 2:** The summary statistics of body weight traits.

Traits	Mean	SD	Median	Min	Max	Skew	Kurtosis	CV (%)
BW80	19.42	4.02	19.30	9.50	34.40	0.245	−0.061	20.73%
BW100	24.55	4.83	24.50	9.78	42.40	0.052	0.073	19.68%
BW120	30.37	5.35	30.30	13.80	49.40	0.013	0.133	17.62%
BW140	36.07	5.74	36.10	19.55	56.30	0.029	0.016	15.91%
BW160	41.87	6.13	41.60	25.10	63.60	0.070	0.122	14.64%
BW180	46.80	6.41	46.80	24.55	70.20	−0.009	0.242	13.70%

**Table 3:** Association analysis between variants of *FGF9* gene and body weight in Hu sheep.

Locus	Genotype	No.	BW80	BW100	BW120	BW140	BW160	BW180
*FGF9*:c.438A>G	GG	471	19.50 ± 0.19	24.61 ± 0.22	30.44 ± 0.25	36.20 ± 0.27	42.05 ± 0.28	46.88 ± 0.30
	GA	478	19.44 ± 0.18	24.52 ± 0.22	30.34 ± 0.25	35.96 ± 0.26	41.75 ± 0.28	46.79 ± 0.29
	AA	118	19.11 ± 0.37	24.58 ± 0.45	30.27 ± 0.49	36.02 ± 0.53	41.64 ± 0.57	46.50 ± 0.59
*FGF9*:c.382-1264C>T	TT	481	19.40 ± 0.18 ^a^	24.56 ± 0.22 ^a^	30.29 ± 0.24 ^a^	36.04 ± 0.26 ^a^	41.54 ± 0.27 ^a^	46.37 ± 0.29 ^ab^
	TC	468	19.33 ± 0.19 ^a^	24.53 ± 0.22 ^a^	30.40 ± 0.25 ^a^	36.15 ± 0.27 ^a^	41.69 ± 0.28 ^a^	46.65 ± 0.30 ^a^
	CC	118	18.36 ± 0.37 ^b^	23.49 ± 0.44 ^b^	29.20 ± 0.48 ^b^	34.92 ± 0.53 ^b^	40.44 ± 0.55 ^b^	45.41 ± 0.59 ^b^

Note: BW, body weight. In the same column, numerical data marked with different lowercase letters indicate significant differences (*p* < 0.05).

**Table 4:** Summary of the epigenetic data (ATAC-seq, H3K4me3, and H3K427ac) for hypophysis tissue in sheep.

Assay	Raw Reads	Clean Reads	Clean Q20 (%)	Clean Q30 (%)	Mapping Rate (%)	RSC	FRiP
ATAC-seq_1	355,833,444	351,268,398	97.52	93.33	99.49	1.56	0.38
ATAC-seq_2	347,033,728	342,040,172	97.35	92.95	99.64	1.87	0.36
H3K4me3_1	71,235,594	66,164,158	98.06	93.51	97.80	1.24	0.79
H3K4me3_2	130,505,626	115,742,448	97.33	91.53	97.40	1.18	0.86
H3K27ac_1	68,907,382	64,771,048	98.49	94.55	98.30	1.42	0.23
H3K27ac_2	91,169,150	84,833,346	98.01	93.19	98.00	1.33	0.68

## Data Availability

The data that support the findings of this study are available from the corresponding author upon reasonable request.

## Supplementary Materials

The following supporting information can be downloaded at https://www.mdpi.com/article/10.3390/ani15162375/s1. Figure S1: Gene frequency and genetic parameters of SNPs for *FGF9* gene in Hu sheep population. (A,B) Gene structure map and SNP location of *FGF9* gene. (C) Genotype frequencies of three SNPs in *FGF9* gene. (D). Genotypic frequency of three SNPs in *FGF9* gene. (E) Genetic parameters of three SNPs in *FGF9* gene. He, heterozygosity; PIC, polymorphism information content; HWE, Hardy–Weinberg equilibrium. Figure S2: Linkage disequilibrium analysis for the SNPs *FGF9*:c.438A>G and *FGF9*:c.393T>C. Table S1: Details of primer sequences used for qRT-PCR and PCR amplification. Table S2: Primer information of each SNP locus. Table S3: Annotation and distribution of epigenetic mark peaks.

## Abbreviations

The following abbreviations are used in this manuscript:

ATAC-seq	Assay for transposase-accessible chromatin-sequencing
BW	Body weight
CV	Coefficient of variation
Cut&Tag	Cleavage under targets and tagmentation
FGF9	Fibroblast growth factor 9
H3K27ac	Histone H3 lysine 27 acetylation
H3K4me3	Histone H3 lysine 4 trimethylation
OCR	Open chromatin region
qRT-PCR	Quantitative real-time PCR
SNPs	Single nucleotide polymorphisms
