# Supplementary material for: A Functional Regulatory Variant of FGF9 Gene Affected the Body Weight in Hu Sheep
Source: Animals (Basel). 2025 Aug 13;15(16):2375. doi: 10.3390/ani15162375 (PMC12382811; doi:10.3390/ani15162375)
Supplement: Supplementary file 1 [file animals-15-02375-s001.zip › animals-3775334-supplementary.pdf]

**Figure**

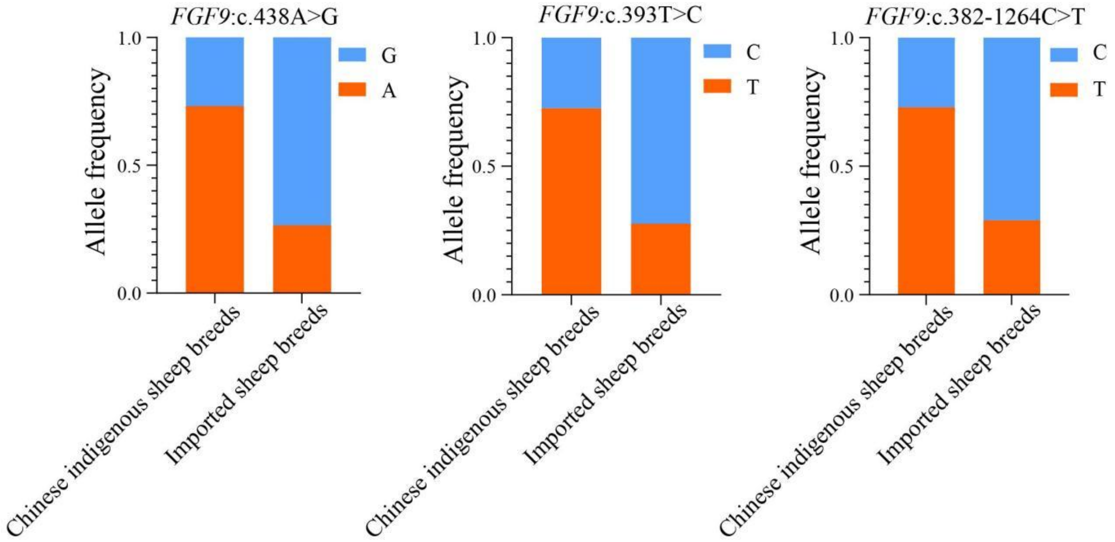


**Figure S1.** Allele frequencies of *FGF9*:c.438A>G, *FGF9*:c.393T>C, and *FGF9*:c.382-1264C>T in Chinese indigenous and imported sheep breeds.

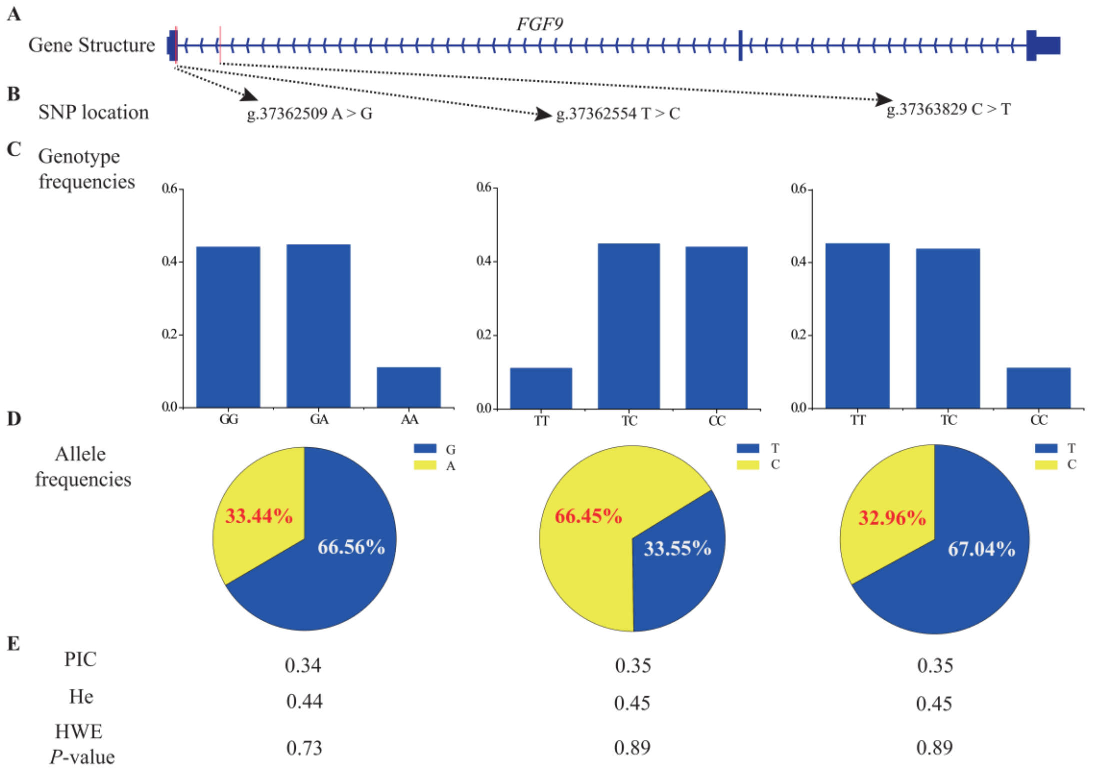


**Figure S2**. Gene frequency and genetic parameters of SNPs for *FGF9* gene in Hu sheep population. A, B. Gene structure map and SNP location of *FGF9* gene. C. Genotype frequencies of three SNPs in FGF9 gene. D. Genotypic frequency of three SNPs in FGF9 gene. E. Genetic parameters of of three SNPs in *FGF9* gene. He,

heterozygosity; PIC, polymorphism information content; HWE, Hardy-Weinberg equilibrium.

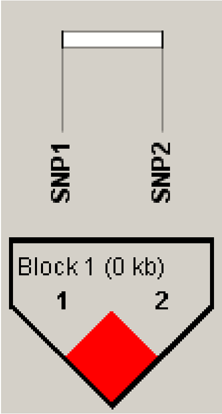


**Figure S3.** Linkage disequilibrium analysis for the SNPs g.37362509 A>G and g.37362554 T>C

**Table**

Table S1 Details of primer sequences used for PCR amplification.

| Primer Name | Primer Sequence (5′-3′) | Annealing Temperature(°C) | Size (bp) |
|---|---|---|---|
| *FGF9*-F1 | TCTCCTCCCCACTCTCTGC | 58 | 624 |
| *FGF9*-R1 | AAATCGGTATTTTCTCACCAG | | |
| *FGF9*-F2 | TGAAAATATTCCCCTTGCTCT | 55 | 365 |
| *FGF9*-R2 | CTCAATCTTTTCCCCTGGT | | |
| *FGF9*-F3 | GAAAACCTACACTCCCCTT | 55 | 562 |
| *FGF9*-R3 | TATATGTATGGCTGAATCCCT | | |
| *FGF9*-F4 | AAAGCCTTCCAGTGTAGACCC | 59 | 615 |
| *FGF9*-R4 | TTGGATTTACCTCGCCGAGT | | |

Table S2 Primer information of each SNP locus.

| Locus | Primer_AlleleX | Primer_AlleleY | Primer_Common |
|---|---|---|---|
| Chr10: g. 37362509 G >A | ACGTTGGATGGCTTTTGTTTTTTGACAGG | ACGTTGGATGATGCTTATACAGGTTGGAGG | AAAAGTTTGAAGAGAACTGGTACAA |
| Chr10: g. 37362554 T > C | ACGTTGGATGATGCTTATACAGGTTGGAGG | ACGTTGGATGGCTTTTGTTTTTTGACAGG | AGGGTCTCTGAAGACACACTCTTG |
| Chr10: g. 37363829 C >T | GAAGGTGACCAAGTTCATGCTCATGAGCCAGCTTCCTGACATC | GAAGGTCGGAGTCAACGGATTTCATGAGCCAGCTTCCTGACATT | TGTGCCTTGGGTGGTTTAAGCGTTT |

Table S3 Annotation and distribution of epigenetic mark peaks.

| Regions | ATAC | | | H3K27ac | | | H3K4me3 | | |
|---|---|---|---|---|---|---|---|---|---|
| | rep1 | rep2 | average | rep1 | rep2 | average | rep1 | rep2 | average |
| Distal Intergenic | 34.33 | 31.62 | 32.975 | 29.21 | 16.14 | 22.675 | 12.56 | 11.45 | 12.005 |
| Intron | 40.77 | 42.39 | 41.58 | 25.36 | 31.82 | 28.59 | 7.53 | 5.72 | 6.625 |
| Promoter | 15.87 | 16.5 | 16.185 | 34.23 | 32.51 | 33.37 | 70.24 | 74.06 | 72.15 |
| Exon | 5.59 | 5.94 | 5.765 | 7.29 | 12.32 | 9.805 | 5.59 | 4.94 | 5.265 |
| Downstream | 1.34 | 1.33 | 1.335 | 1.27 | 1.4 | 1.335 | 1.02 | 0.83 | 0.925 |
| 3' UTR | 1.83 | 1.91 | 1.87 | 2.27 | 4.82 | 3.545 | 2.56 | 2.5 | 2.53 |
| 5' UTR | 0.26 | 0.31 | 0.285 | 0.38 | 0.99 | 0.685 | 0.51 | 0.5 | 0.505 |
